# Shortcomings of Administrative Data to Derive Preventive Strategies for Inhospital Drug-Induced Acute Kidney Failure—Insights from Patient Record Analysis

**DOI:** 10.3390/jcm11154285

**Published:** 2022-07-23

**Authors:** Stefanie Amelung, David Czock, Markus Thalheimer, Torsten Hoppe-Tichy, Walter E. Haefeli, Hanna M. Seidling

**Affiliations:** 1 Department of Clinical Pharmacology and Pharmacoepidemiology, Heidelberg University Hospital, Im Neuenheimer Feld 410, 69120 Heidelberg, Germany; stefanie.amelung@med.uni-heidelberg.de (S.A.); david.czock@med.uni-heidelberg.de (D.C.); walter-emil.haefeli@med.uni-heidelberg.de (W.E.H.); 2 Cooperation Unit Clinical Pharmacy, Heidelberg University Hospital, Im Neuenheimer Feld 410, 69120 Heidelberg, Germany; torsten.hoppe-tichy@med.uni-heidelberg.de; 3 Hospital Pharmacy, Heidelberg University Hospital, Im Neuenheimer Feld 670, 69120 Heidelberg, Germany; 4 Department of Quality Management and Medical Controlling, Heidelberg University Hospital, Im Neuenheimer Feld 672, 69120 Heidelberg, Germany; markus.thalheimer@med.uni-heidelberg.de

**Keywords:** International Classification of Diseases, inpatients, adverse drug events, preventability, acute kidney injury

## Abstract

Structured analyses of hospital administrative data may detect potentially preventable adverse drug events (ADE) and therefore are considered promising sources to prevent future harm and estimate cost savings. Whether results of these analyses indeed correspond to ADE that may be preventable in clinical routines needs to be verified. We exemplarily screened all adult inpatients admitted to a German University Hospital (n = 54,032) for International Classification of Diseases-10th revision (ICD-10) diagnoses coding for drug-induced kidney injury (AKI). In a retrospective chart review, we checked the coded adverse events (AE) for inhospital occurrence, causality to drug exposure, and preventability in all identified cases and calculated positive predictive values (ppv). We identified 69 inpatient cases of whom 41 cases (59.4%) experienced the AE in the hospital (ppv-range 0.43–0.80). Causality assessment revealed a rather likely causal relationship between AE and drug exposure in 11 cases (15.9, 11/69, ppv-range 0.17–0.22) whereby preventability measures could be postulated for seven cases (10.1%, 7/69). Focusing on drug-induced AKI, this study exemplarily underlines that ICD-10-code-based ADE prevention efforts are quite limited due to the small identification rate and its high proportion of primarily outpatient events. Furthermore, causality assessment revealed that cases are often too complex to benefit from generic prevention strategies. Thus, ICD-10-code-based calculations might overestimate patient harm and economic losses.

## 1. Introduction

Clinical administrative data (CAD) are a widely used data source for research questions, including the identification of adverse drug events (ADE) by using ICD-9 or ICD-10 codes (International Statistical Classification of Diseases and Related Health Problems) [1,2,3,4,5,6,7,8]. Often, identified ADE are then correlated with prolonged length of hospital stay [9,10,11] or, congruently, with substantially higher treatment costs compared to patients without identified ADE, as shown for German or Canadian inpatients [9,12]. The calculated additional costs, therefore, seem to indicate potential savings, presuming that ADE could have been avoided [9,12].

Indeed, as several studies show, inhospital ADE load is high and has a crucial impact on patient outcomes and economic burden [10,11,12]. However, the German coding system does not precisely specify when the coded event occurs, i.e., before or during the hospital stay [13], and whether the coded event could have been preventable or not. Thus, in order to estimate the potential cost savings and preventable patient harm of ADE identified by ICD-10-codes from a hospital’s perspective, the amount of actual inpatient and preventable ADE needs to be verified.

In a previous analysis, we compiled a list of ICD-10 codes referring to ADE that were suspected of typically occurring during the hospital stay and might be preventable. We performed propensity score analyses in order to estimate the clinical relevance of the respective codes with respect to the prolongation of the length of stay of the affected patients [10]. Based on these analyses, ICD-10 codes coding for drug-induced renal failure appeared clinically relevant because of the frequency of coded events and the prolonged inhospital stay of affected patients. Mechanistic considerations about possible causes, and the opinion of clinicians confronted with affected patients, led us to rate the causes leading to drug-induced renal failure as potentially preventable. These ratings, however, were performed without access to individual patient records [10]. In order to validate the usability of these codes, a clinical verification of the exact cases is needed.

The aims of this study were, therefore, (i) to confirm the actual inhospital occurrence of the coded adverse events (AE), (ii) to assess the causality between suspected drugs and the AE, and (iii) to analyse the causes and conditions leading to inhospital drug-induced renal failure in order to determine whether common prevention strategies could have avoided them.

## 2. Methods

### 2.1. Study Design and Setting

All adult inpatients admitted to Heidelberg University Hospital, Germany, in 2012, whose stays were covered by German health insurance (n = 54,032), were screened for predefined ICD-10 codes that are typically used for coding drug-induced nephropathy (N14.1 or N14.2) or postprocedural renal failure (N99.0, see also Table 1). The validity of these codes, previously assessed as having occurred in the hospital and being potentially preventable ADE [10], was verified by manual patient record analysis. 

The study protocol was approved by the responsible Ethics Committee of the Medical Faculty of Heidelberg University.

### 2.2. Validity of ICD-10 Codes for Inhospital Renal Failure

Following the KDIGO guidelines, renal failure was defined as acute kidney injury (AKI) if any of the following conditions were present: an increase in serum creatinine (SCr) by ≥0.3 mg/dl within 48 h; an increase in SCr to ≥1.5 times baseline, which is known or presumed to have occurred within the prior seven days, or a urine volume <0.5 mL/kg/h for six hours [14]. Inhospital occurrence of AKI was confirmed by analyzing laboratory test results of SCr or documented urine volume throughout the hospital stay.

### 2.3. Causality Assessment and Conceivable Prevention Strategies

For causality assessment, all relevant information was summarized in a standardized form. We collected information about the patient (i.e., age, sex, known diagnoses at admission, new diagnoses at discharge, and the comorbidity index PCCL (patient clinical complexity level, a medical severity classification whose value depends on all coded secondary diagnoses [15])), the patient’s hospital stay (date of admission and discharge, changes in wards, and discharging clinic), drug treatment (drugs taken before and during the hospital stay and at discharge with dosages and routes of administration, and start and end date), the AE (related ICD-10 code, medical history, thereby explicitly checking for potential contributing factors, such as nonsteroidal anti-inflammatory drugs, iodine-containing contrast media, low blood pressure, and/or severe infections), renal replacement procedures, and further clinical course and complications.

We used the updated Bégaud score for the causality assessment of AE to drug exposure [16]. Briefly, this method results in a numerical score, which can be divided into three parts: an informativeness score (three items: NI0 to NI2), reflecting the level of information available to assess the drug–AE combination, bibliographic criteria (four items: B1 to B4) displaying the knowledge about the assessed drug–AE combination in the scientific literature, and the intrinsic imputability score. The latter score consists of seven items (I0 to I6) combined from two different criteria: chronological criteria (time to onset, discontinuation, and re-administration of a drug, C0 to C3) and semiological criteria (S0 to S3), which describe the signs and symptoms that suggest the drug may have caused the AE, predisposing factors, and results of specific and reliable investigations, but also considers the search for other non-drug etiologies [16]. Predisposing factors for AKI have been extracted from the literature [17,18,19] (as tabulated in online-only Supplementary Materials Table S1). Two reviewers (SA and DC) independently assessed the extracted information for causality assessment. If no temporal link of any drug to the AE could be found (C0) or obvious non-drug causes could explain the AKI (S0), cases were classified as not drug-induced (I0) and excluded from further preventability assessment. In order to simplify the comparison of the reviewers’ ratings on the seven intrinsic imputability scores, we defined drugs as rather unlikely causative if the intrinsic imputability score ranged from I0 to I3, and drugs with a score of I4–I6 as rather likely causative to the AE. The first three cases were used to pilot the causality assessment and to enable the definition of exclusion criteria for patient records not suitable to establish preventive measures. The pilot cases were included in all further analyses.

We have adapted techniques of Root Cause Analysis to our data set (e.g., using a formal protocol, focusing on organizational factors, determining the chronology of events, and identifying potentially contributing factors) [20,21] in order to identify the causes of AE in all cases where a drug was considered as rather likely causative to the AE (I4–I6). On this basis, the two reviewers attempted to derive solution-oriented, common prevention strategies to minimize recurrence. 

### 2.4. Statistical Analyses

Metric and normally distributed variables were reported as mean ± standard deviation (SD), range, and median. Categorical variables were presented as frequency and percentage. To quantify the validity of the three previously assessed ICD-10 codes (Table 1), the positive predictive value (ppv) was calculated. Because the negative predicted condition of all analyzed codes was previously set to zero in all cases, we could not calculate sensitivity rates, specificity rates, or negative predictive values. For all cases included in the causality assessment, the kappa test was calculated to quantify the interrater reliability of the causality assessment between both reviewers. Subsequently, consensus was found in the case of diverging judgments.

Data management was performed with MS Access 2010, data analysis by MS Excel 2010 (both Microsoft, Redmont, WA, USA), SPSS 22.0, 2013 (IBM, Armonk, NY, USA), and GraphPad Prism 5.01, 2007 (GraphPad Software, La Jolla, CA, USA).

## 3. Results

### 3.1. Patient Characteristics

The records of all 69 identified inpatients (33% female) with ICD-10 codes coding for drug-induced renal failure were analyzed. The mean patient age was 62 years and the mean length of stay was 22.1 days. Ten patients exceeded the maximum hospital stay that is fully reimbursed by insurance companies (Table 2).

### 3.2. Validity of ICD-10 Codes for Inhospital AKI

Based on the course of their SCr values, 41 patients developed an inhospital AKI while the remaining 28 patients turned out to have a known history of renal failure already at admission. The ppv of all codes for an inhospital event was 0.59, and was highest for N99.0 (ppv = 0.8, see Table 1). Following our exclusion criteria, we excluded ten patients admitted for stem cell transplantation, because in these patients kidney injury occurs very often and is usually multi-factorial (including severe infections, calcineurin inhibitor toxicity, and thrombotic microangiopathy) and, therefore, not suitable for implementation of preventive strategies. This yielded 31 cases with an inhospital AE eligible for causality assessment.

### 3.3. Causality Assessment and Preventive Strategies

Both reviewers independently screened all 31 inpatients eligible for causality assessment and their 741 drug prescriptions. The two reviewers agreed on the causality assessment in 25 of the 31 patient cases, with κ = 0.62, which is rated as good interrater reliability [22], and identified the same drugs eligible for causality assessment in 41.9% (13/31) of the evaluated cases. The informativeness score (NI) was NI2 in all 31 inpatient cases, meaning that for each drug–AE pair all relevant information was available to assess causality. The bibliographic criteria of all causality assessments met score B4, describing the AE as listed in the summary of product characteristics. In 711 of the 741 drug prescriptions, either no temporal link existed (C0, 290 drugs), or the drugs were not known to be nephrotoxic, or predisposing factors or non-drug causes could have induced non-drug-induced AKI (S0, 421 drugs). For eleven inpatients, no causal relationship between any of the given drugs and the AE was present (I0, 35.5%, 11/31). Reasons more likely to have induced renal failure in these cases were perioperative AKI (n = 8), sepsis and multi-organ dysfunction (n = 2), and bilateral nephrectomy (n = 1). Reasons for perioperative AKI may include drug-induced hypotension, but a single causative drug can usually not be identified.

**Figure 1:**
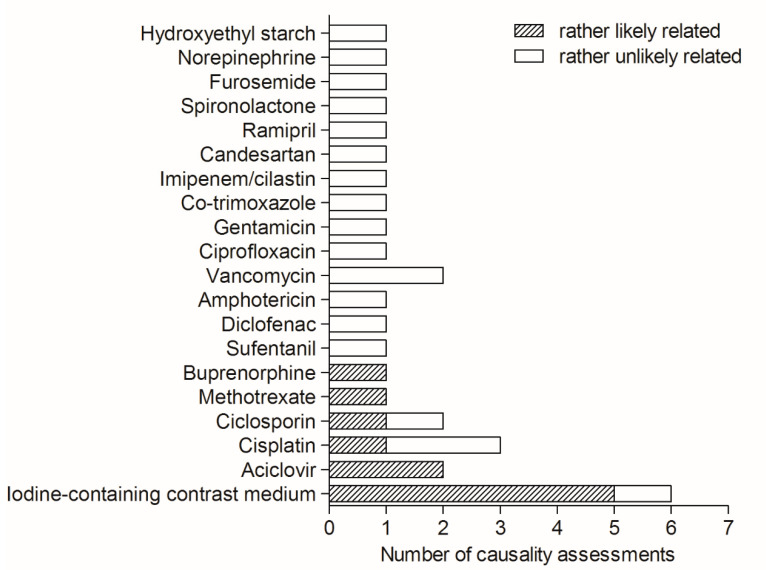
Causality assessment for adverse events and involved drugs: Outcome of the 30 causality assessments in 20 patients after consensus finding. Twenty different drugs were involved. A rather likely causal relationship to the adverse event was found for six different drugs, corresponding to eleven inpatient cases. Multiple causality assessments for the same patient occurred in four patients.

Conversely, in 20 of the 31 inpatient cases and 30 drug prescriptions, the causality categories differed from C0 or S0, see also Figure 1. After consensus finding, the reviewers agreed on eleven inpatient cases out of all cases eligible for causality assessment (35.5%, 11/31) where a drug was considered as rather likely causative for the AE (I4–I6). The involved drugs were iodine-containing contrast media (n = 5), aciclovir (n = 2), and buprenorphine, cisplatin, ciclosporin, and high-dose methotrexate (n = 1, each, Figure 1). The ppv of all codes for an inhospital ADE was thus 0.19 and highest for N14.1 (ppv = 0.22, see Table 1).

Out of these eleven cases, we were able to develop potential prevention strategies for two of the six involved drugs (aciclovir and iodine-containing contrast media), corresponding to seven inpatient cases (23%, Table 3).

Preventive strategies could be hardly elaborated for the four cases with buprenorphine, methotrexate, cisplatin, or ciclosporin. Buprenorphine triggered hypotension, subsequently lowered renal perfusion, and was considered a previously unknown intolerance of this patient and therefore rated as not preventable. In the latter three cases recommended precautions (e.g., sufficient hydration in all patients, urine-pH monitoring, and urine alkalization during methotrexate) were already set in place as far as assessable from the documentation in the chart. The ppv for preventable inhospital AKI was thus 0.10 for all codes together and highest for N14.1 (ppv of 0.22, see Table 1).

## 4. Discussion

Our evaluation illustrates some major shortcomings of the ICD-10 code triggered identification of inhospital drug-induced AKI and preventability predictions based on routine data analyses. First, the actual AE only inconsistently started during the hospital stay: in about 40% of the cases, it was already present at admission, which is in line with another German study, using ADE codes for identification [28]. This indicates that preventive measures, if available, might have been required before hospital admission. 

Second, and even more striking, causality assessments using the updated Bégaud score only rarely identified drugs or medication errors as a convincing trigger for the AE (ppv = 0.19, range 0–0.22), defined as an intrinsic imputability score of at least I4. In many cases, the AE was not likely caused by a drug or at least not by a drug alone. Most often, patients had severe underlying diseases and other individual contributing risk factors suggesting that the causes for AKI were likely multi-factorial, thus precluding the deduction of a straightforward and generic prevention strategies. Another study reported a much higher ppv of 0.79 for ICD-10-codes coding for inhospital ADE but based the causality assessment on expert opinion alone, not considering contributing factors or other non-drug causes for respective AE [29]. Hence, the different methodology prevents study comparison.

Furthermore, the prevalence of drug-induced AKI coded with the respective ICD-10 code was lower compared to prospective studies assessing AKI in inpatients. Typically, the incidence of drug-induced AKI concerned 0.49–1.3% of the patients [30,31], whereas using ICD-10 codes from routine data yielded more than six times lower incidence rates (0.08%, 41/54, 032). Another study examining hospitalizations due to ADE showed similar results, with identification rates by ICD-10 codes more than five times lower compared to prospective ADE identification [31]. With our causality assessment results and the mentioned substantial underreporting in mind, ADE detection based on administrative data tends to identify the more severe cases. This is in line with a study investigating ICD-10 coding of the different stages of AKI [32]. This excludes less severe cases from causality assessment and analysis of preventive efforts that might influence the rate of potentially preventable inpatient cases. Conversely, for only seven of the investigated 69 inpatient cases (10.1%, 7/69) and two drugs involved, were we able to propose common prevention strategies, e.g., the stringent use of fluid-balancing protocols in patients with, e.g., iodine-containing contrast media, aciclovir, or methotrexate. Preventability therefore might not be a characteristic of a certain diagnosis code, but rather be patient-related and setting-specific and, hence, depends on context information.

Our data set did not allow calculating sensitivity rates for our ICD-10 codes indicative for ADE. Studies that report sensitivity rates for ICD-10 codes showed rather low sensitivity rates of ICD-10 codes indicative for an ADE (6.8%) [3], or ICD-10 codes indicative for non-drug induced AKI (11.7–17.2%) [33,34]. This stresses the limited and highly variable accuracy of ICD-10 codes, even in the US American setting, where ICD-9 codes for healthcare-associated infections are used for internal and external benchmarking processes [35]. In addition and in contrast to the German coding system, inhospital occurrence of diagnoses can be coded within the administrative database in the US, Canadian, and Australian coding systems, which might influence sensitivity rates [7,36,37].

Thus, our study clearly underlines the fact that CAD-driven ADE analysis may not be a useful tool to derive potential cost savings as the ADE might not be easily preventable in the first place. Nevertheless, there are several studies calculating clinical and economic risks on the basis of ICD-10 codes, among others there are also codes for renal failure [5,9,10,12]. Based on our results, it can be assumed that these studies may have overestimated the potential of preventable ADE within the group of identified AE. We therefore strongly recommend an appropriate causality assessment or the use of fitting ppv of ICD-10 codes indicative for ADE. In our study, we used the updated Bégaud score [16]. In comparison to most other algorithms, it takes the literature related to the drug and adverse reactions into account, and combines two different decision tables (chronological and semiological criteria, which also take laboratory values into account) into one intrinsic causality score. For causality assessment, there is no gold standard algorithm [38]. However, in order to compare our findings as well as possible to the existing literature, we chose this validated, sensitive, and specific method [39,40], which is widely used, e.g. by the French pharmacovigilance system [41,42]. For fitting ppv, our study adds a different and likely more realistic ppv on ICD-10 codes coding for drug-induced AKI. It can thus help to soundly approximate calculations of harm and economic loss based on administrative data and help (re)direct prevention efforts.

The applied study design has several limitations. First of all, the inevitably retrospective design partly impeded the preventability assessment as, for instance, no interviews of involved health care professionals or patients were possible. Hence, proposed measures might have even been set in place but not documented in detail. Thus, postulated preventability and proposed prevention strategies need to be seen as first suggestions, and then would need prospective verification in routine care. Second, while incidence rates of ICD-10 codes indicative for ADE are in line with other studies, we report results of a single-center analysis in a university hospital setting. Coding habits may have differed between our various departments, and such differences may be of greater importance between different centers with various administrative databases, patient records, and clinical decision support systems, which all might influence the incidence rates.

## 5. Conclusions

Within the limitations of a single center study, this study emphasises that ADE analyses based on hospital discharge data carry the risk of overestimating potential cost savings by generalizing treatment-related problems and ignoring patient- and context-relevant factors. Moreover, it also outlines that the few identified ADE are often too complex to be easily caught by the few and generic prevention measures. To more reliably estimate preventable inhospital AE and its economic burden on the basis of ICD-10-codes, we strongly recommend the use of appropriate causality assessment or fitting ppv of the respective ICD-10 codes. For this, our study adds another, probably more realistic ppv of ICD-10 codes coding for inhospital and drug-induced AKI, showing that administrative data-based calculations of patient harm and economic loss are insufficient.

## Figures and Tables

**Table 1:** ICD-10 codes used for drug-induced nephropathy or postprocedural renal failure and corresponding positive predictive value (ppv) for an inhospital, drug-induced, and potentially preventable event.

**ICD-10 Code**	**Code Description**	**Patient Cases (n)**	**N with an Inhospital Event**	Ppv for an Inhospital Event *	N = 10 excluded because of stem cell transplantation	**N**	**N with an Inhospital, Drug-Related Event**	Ppv for an Inhospital, Drug-Induced Event *	**N with an Inhospital, Drug-Related, Potentially Preventable Event**	Ppv for an Inhospital, Drug-Induced and Potentially Preventable Event *
N14.1	Nephropathy induced by other drugs, medicaments and biological substances	21	11	0.52		18	4	0.22	4	0.22
N14.2	Nephropathy induced by unspecified drug, medicament, or biological substance	23	10	0.43		18	3	0.17	0	0.00
N99.0	Postprocedural renal failure	25	20	0.80		23	4	0.17	2	0.09
Total		69	41	0.59		59	11	0.19	6	0.10

* While the ppv for an inhospital event was calculated based on all 69 patient cases, the ppv of drug-induced and preventable events was based on only 59 patient cases, excluding 10 patient cases admitted for stem cell transplantation.

**Table 2:** Patient characteristics of the 69 inpatients with one of the ICD-10 codes coding for drug-induced nephropathy or postprocedural renal failure.

Patient Characteristic	N (%) or Mean ± SD [Min-Median-Max]
All, n	69 (100)
Men, n	46 (67)
Women, n	23 (33)
Age, y	62 ± 15.6 [23-63-94]
Patients aged ≥ 65 y, n	32
PCCL	3 [0-4-4]
ICD-10 codes/patient, n	15 ± 9.2 [2-14-45]
Length of stay, d	22.1 ± 18.3 [1-17-88]
Patients exceeding length of stay, n	10 (14)
Exceedance of length of stay, d	9.7 ± 15.9 [1-52]

PCCL: Patient Clinical Complexity Level, a co-morbidity marker whose value depends on all coded secondary diagnoses, ranging from 0 (no co-morbidity) to 4 (highest co-morbidity) [15].

**Table 3:** Inpatient cases with potentially preventable ADE for whom possible prevention strategies could be postulated.

Case #, Age (y), Sex	Involved Drug	Risk Factors [17,18,19]	Prevention Strategy
#1, 74, female	Intravenous aciclovir	Older age, Diabetes mellitus type 2, Severe underlying malignant disease (diffuse large B cell lymphoma)	Adequate fluid intake, fluid-balancing protocols, slow infusion rate over one hour of intravenous aciclovir [17,23]
#2, 83, male	Intravenous aciclovir	Older age, Male gender, Preexisting chronic kidney disease grade II/IIIa, Cardiac disease (mild heart failure), Dehydration	
#3, 74, male	Iodine-containing contrast agent (unknown substance)	Older age, Male gender	Adequate fluid intake, fluid-balancing protocols, use of iso-osmolar or low-osmolar preparations in lowest possible doses [24,25,26,27]
#4, 81, male	Iodine-containing contrast agent (iomeprol, low-osmolar)	Older age, Male gender, Arterial hypertension, Cardiac disease (NSTEMI, coronary heart disease), Diabetes mellitus type 2, Concomitant infection (urosepsis)	
#5, 75, male	Iodine-containing contrast agent (unknown substance)	Older age, Male gender, Preexisting chronic kidney disease grade III, Diabetes mellitus type 2, Concomitant infection (pneumonia)	
#6, 94, female	Iodine-containing contrast agent (unknown substance)	Older age, Heart failure, Preexisting chronic kidney disease grade III/IV, Concomitant infection (severe bacterial infection)	
#7, 49, male	Iodine-containing contrast agent (unknown substance)	Diabetes mellitus type 2, Male gender	

## Data Availability

The datasets generated and/or analyzed during the current study are not publicly available due to local privacy policies.

## Supplementary Materials

The following supporting information can be downloaded at: https://www.mdpi.com/article/10.3390/jcm11154285/s1, Table S1: Non-drug related risk factors associated with the development of acute renal injury.

## Abbreviations

ADE	adverse drug events
CAD	clinical administrative data
ICD-10	International Classification of Diseases-10th revision
AE	adverse events
AKI	acute kidney injury
SCr	serum creatinine
SD	standard deviation
ppv	positive predictive value
