# Supplementary material for: Shortcomings of Administrative Data to Derive Preventive Strategies for Inhospital Drug-Induced Acute Kidney Failure—Insights from Patient Record Analysis
Source: J Clin Med. 2022 Jul 23;11(15):4285. doi: 10.3390/jcm11154285 (PMC9330816; doi:10.3390/jcm11154285)
Supplement: Supplementary file 1 [file jcm-11-04285-s001.zip › jcm-1791426-supplementary.pdf]

Shortcomings of administrative data to derive preventive strategies for inhospital drug-induced acute kidney failure – insights from patient record analysis

Journal of Clinical Medicine

Stefanie Amelung[1,2,3] (Stefanie.Amelung@med.uni-heidelberg.de), David Czock[1] (David.Czock@med.uni-heidelberg.de), Markus Thalheimer[4] (Markus.Thalheimer@med.uni-heidelberg.de), Torsten Hoppe-Tichy[2,3] (Torsten.Hoppe-Tichy@med.uni-heidelberg.de), Walter E. Haefeli[1,2] (Walter-Emil.Haefeli@med.uni-heidelberg.de), Hanna M. Seidling*[1,2] (Hanna.Seidling@med.uni-heidelberg.de)

Affiliations:

[1] Department of Clinical Pharmacology and Pharmacoepidemiology, Heidelberg University Hospital, Im Neuenheimer Feld 410, 69120 Heidelberg, Germany,

[2] Cooperation Unit Clinical Pharmacy, Heidelberg University Hospital, Im Neuenheimer Feld 410, 69120 Heidelberg, Germany,

[3] Hospital Pharmacy, Heidelberg University Hospital, Im Neuenheimer Feld 670, 69120 Heidelberg, Germany,

[4] Department of Quality Management and Medical Controlling, Heidelberg University Hospital, Im Neuenheimer Feld 672, 69120 Heidelberg, Germany

Corresponding author:

Prof. Dr. sc. hum. Hanna Seidling
Cooperation Unit Clinical Pharmacy
Department of Clinical Pharmacology and Pharmacoepidemiology
Heidelberg University Hospital
Im Neuenheimer Feld 410
69120 Heidelberg
Phone: +49 6221 5638736
Fax: +49 6221 564642
Hanna.Seidling@med.uni-heidelberg.de

Non-drug risk factors for acute renal injury

Table S1 Non-drug related risk factors associated with the development of acute renal injury.

Risk factor	References
Age (> 60 years old)	[1–3]
Male sex	[2]
Obesity	[3]
Jaundice	[3]
Infection	[2]
Sepsis	[1,2]
Multiple organ failure	[2]
Shock	[1]
Hypovolemia	[1]
Cardiovascular failure	[2]
Respiratory failure	[2]
Mechanical ventilation	[2]
Cardiac surgery	[1,2]
Rhabdomyolysis	[1]
Preexisting chronic disease	[2]
Preexisting renal disease	[1-3]
Diabetes	[1-3]

References

1. Brochard L, Abroug F, Brenner M, Broccard AF, Danner RL, Ferrer M, Laghi F, Magder S, Papazian L, Pelosi P, Polderman KH, Failure AEESSAHCoAR (2010) An Official ATS/ERS/ESICM/SCCM/SRLF Statement: Prevention and Management of Acute Renal Failure in the ICU Patient: an international consensus conference in intensive care medicine. American journal of respiratory and critical care medicine 181 (10):1128-1155. doi:10.1164/rccm.200711-1664ST
2. Pruchnicki MC, Dasta JF (2002) Acute renal failure in hospitalized patients: part I. The Annals of pharmacotherapy 36 (7-8):1261-1267. doi:10.1345/aph.1A339
3. Singri N, Ahya SN, Levin ML (2003) Acute renal failure. JAMA : the journal of the American Medical Association 289 (6):747-751
